# Nuclear β-catenin expression is closely related to ulcerative growth of colorectal carcinoma

**DOI:** 10.1038/sj.bjc.6600214

**Published:** 2002-04-08

**Authors:** J M Chiang, YH Wu Chou, T C Chen, K F Ng, J L Lin

**Affiliations:** Division of Colon and Rectal Surgery, Human Molecular Genetics Laboratory, Chang Gung Memorial Hospital, 199 Tung Hwa North Road, Taipei, Taiwan 333; Department of Pathology, Chang Gung Memorial Hospital, 199 Tung Hwa North Road, Taipei, Taiwan 333

**Keywords:** β-catenin, colorectal cancer, polypoid tumour, ulcerative tumour, carcinogenesis

## Abstract

Although most colorectal cancer develops based on the adenoma–adenocarcinoma sequence, morphologically, colorectal cancer is not a homogeneous disease entity. Generally, there are two distinct morphological types: polypoid and ulcerative colorectal tumours. Previous studies have demonstrated that K-*ras* codon 12 mutations are preferentially associated with polypoid growth of colorectal cancer; however, little is known about the molecular mechanism that determines ulcerative growth of colorectal cancer. β-catenin complex plays a critical role both in tumorigenesis and morphogenesis. We examined the differential expression of β-catenin and its related factors among different types of colorectal cancer in order to determine any relationship with gross tumour morphology. Immunohistochemical staining of β-catenin, E-cadherin and MMP-7 was performed on 51 tumours, including 26 polypoid tumours and 25 ulcerative tumours. Protein truncation tests and single-strand conformational polymorphism for mutation of the adenomatous polyposis coli tumour suppressor gene, as well as single-strand conformational polymorphism for the mutation of β-catenin exon 3 were also done. Nuclear expression of β-catenin was observed in 18 out of 25 (72%) cases of ulcerative colorectal cancer and seven out of 26 (26.9%) cases of polypoid colorectal cancer. A significant relationship of nuclear β-catenin expression with ulcerative colorectal cancer was found (*P*<0.001). However, this finding was independent of adenomatous polyposis coli tumour suppressor gene mutation and E-cadherin expression. Together with previous data, we propose that different combinations of genetic alterations may underlie different morphological types of colorectal cancer. These findings should be taken into consideration whenever developing a new genetic diagnosis or therapy for colorectal cancer.

*British Journal of Cancer* (2002) **86**, 1124–1129. DOI: 10.1038/sj/bjc/6600214 www.bjcancer.com

© 2002 Cancer Research UK

## 

Colorectal carcinogenesis involves a multistep progression of genetic mutations. Based on the adenoma–carcinoma sequence, much research has focused on mutation detection; sequential genetic alterations have been illustrated as a linear process (Fearon and Vogelstein, 1990; Kinzler and Vogelstein, 1996). Although this approach represents a well-known paradigm for the sequential development of cancer driven by the accumulation of genetic defects, more and more cases of carcinogenesis have been reported in contrast to the linear and clonal development of cancer (Sedivy *et al*, 2000). Recently, a non-linear, chaodynamic model of carcinogenesis has been suggested by others. In this model, genetic instability among cells produces a tremendous and chaotic diversity that may lead to cancer (Coffey, 1998). Furthermore, from a holistic point of view, not only genetic events may drive the onset of cancer. In biology, deterministic and non-deterministic phenomena co-exist. Clinically, in terms of gross morphology, colorectal cancer (CRC) is not a homogeneous disease entity (Kudo, 1993; Hamilton, 1995; Kato *et al*, 1997). In general, there are two common but distinct morphological types, namely polypoid and ulcerative colorectal tumours (Hamilton, 1995). These are distinguished by different gross appearances: either an exophytic or endophytic growth pattern (Figure 1

**Figure 1:**
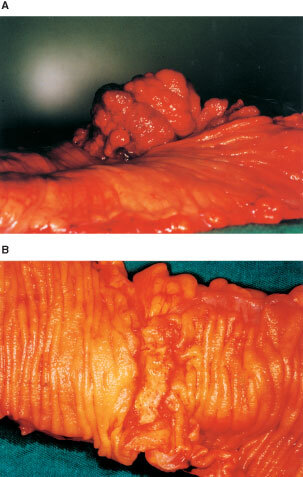
(**A**) polypoid tumour; (**B**) ulcerative tumour.

). Polypoid tumours always show distinguished protruding or exophytic growth towards the bowel lumen (Figure 1A). Ulcerative tumours have an ulcer that usually exhibits endophytic growth toward the bowel wall itself; thus the tumour's floor may be below the surface of the surrounding mucosa (Figure 1B). These divergent morphological features lead us to question whether different genetic or different combinations of genetic alterations are what are involved in colorectal carcinogenesis.

In the model proposed by Vogelstein *et al* (1988), adenomatous *polyposis coli* (APC) tumour suppressor gene mutation is found in the earliest stage, before proceeding to K-*ras* mutation as well as to other tumour suppressor genes, such as p53 and DCC. Paralleling increases in adenoma size and grade of dysplasia ensue during colorectal tumour progression. However, nothing has been described on whether different genetic alterations occurred between these two distinct types of colorectal cancer during multistep carcinogenesis. Although data from synchronous adenoma and carcinoma analyses emphasised that the accumulation of genetic alterations is more important than their order (Fearon and Vogelstein, 1990; Kinzler and Vogelstein, 1996), some researchers are still interested in establishing an ideal linear process. Nonetheless, there are reports indicating that the significantly low frequency of K-*ras* mutation is associated with superficial or non-polypoid type colorectal adenoma or carcinoma (Yamagata *et al*, 1994, 1995). Others and we have further demonstrated significant correlation between polypoid growth of CRC and K-*ras* codon 12 mutation (Chiang *et al*, 1998). However, with regard to the ulcerative type of colorectal cancer, this remains poorly understood. We investigated the expression of β-catenin, which plays an important role in the morphogenesis and carcinogenesis of colorectal cancer (Gumbiner, 1996). Apart from its involvement in cell adhesion and the Wingless/Wnt signaling pathway, β-catenin may play a direct role in colorectal carcinogenesis because it binds the products of the APC tumour suppressor gene. When APC is mutated, which occurs in up to 80% of colorectal cancer, β-catenin accumulates and translocates to the nucleus, where it binds the transcription factors of the TCF/LEF gene family and activates the expression of target genes (Korinek *et al*, 1997).

We attempted to detect if any differential expression of β-catenin was related to different morphological growth patterns in colorectal carcinomas. Factors related to altered β-catenin expression including E-cadherin expression, mutations of the APC tumour suppressor gene or the β-catenin gene itself were also investigated. Materialising matrix metalloproteinase-7 (MMP-7) expression, one of the downstream targets of β-catenin, was also analysed in relation to β-catenin expression.

## MATERALS AND METHODS

### Sample collection

We collected 51 primary colorectal carcinoma tissue samples from sporadic colorectal cancer patients who underwent colectomies at Chang Gung Memorial Hospital (CGMH). All samples were collected immediately after resection and stored in a −80°C freezer. Normal mucosa samples were removed at the same time from sites about 10 cm from each tumour. Whole tumour specimens were then prepared for routine histopathological examination. Formalin-fixed, paraffin-embedded colorectal carcinoma tissue samples were preserved in the tissue archives of the Pathology Department at CGMH.

Detailed morphological descriptions, histopathological data and clinical data were obtained for each case from the Cancer Registry of the Department of Colorectal Surgery at CGMH.

Tumours with exophytic cauliflower-like appearances with or without a very shallow ulcer only, and with a height exceeding half their diameter, were classified as polypoid (Figure 1A). Tumours within depressed ulcers with or without very low elevated edges, and showing endophytic growth, were classified as ulcerative (Figure 1B).

### Immunohistochemistry

Standard immunohistochemical detection with minor modifications was performed on sections from the archival, paraffin-embedded tissue to detect E-cadherin, β-catenin and matrilysin proteins.

Five-micron sections mounted onto slides were deparaffinised and rehydrated in graded alcohols and distilled water. Endogenous peroxidase activity was inhibited by incubation with 3% hydrogen peroxide in methanol for 20 min. Antigen retrieval was done by microwaving at high power for two cycles of 5 min each, with a 10-min break between cycles in citrate buffer at pH 6.0. Non-specific binding of secondary antibodies was blocked by incubation in 10% normal rabbit serum.

Incubations with primary antibodies were done at 37°C for 120 min for anti E-cadherin (Transduction Laboratories, Lexington, KY, USA) at 100× dilution, 60 min at 37°C for anti-β-catenin (Transduction Laboratories) at 200× dilution and 1 : 300 for MMP-7 (Chemicon, Hofhem, Germany). After three washes with phosphate buffered saline (PBS), the slides were incubated with biotinylated antimouse immunoglobulin and stained using the Ultra Tech Detection System Kit (Immunotech, Cedex, France). 3,3′ Diaminobenzidine (DAB) was used as the chromogen. The slides were counterstained with hematoxylin and dehydrated in graded alcohols, air-dried and mounted using a resin-based mounting medium (Immunotech) under coverslips.

### Stain interpretation

**Figure 2:**
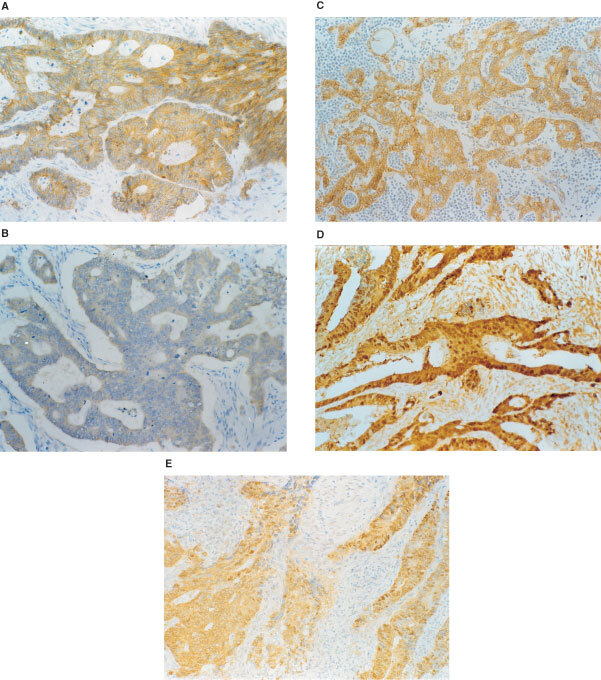
Representative immunohistochemical staining of E-cadherin and β-catenin in resected colon specimens from sporadic CRC: (**A**) E-cadherin and (**C**) β-catenin are mainly localised at the membranes of the cell-to-cell borders (preserved, paraffin-embedded colorectal carcinoma); (**B**) loss of membrane staining for E-cadherin. Both (**D**) and (**E**) show a loss of membrane staining of β-catenin with reciprocal change in nuclear β-catenin expression. (**D**) Diffuse, widespread nuclear immunostaining for β-catenin is profound in colon carcinoma cells, while (**E**) shows focal, clustered nuclear β-catenin expression. **A**–**E**: ×400.

Slides were independently examined by two experienced observers (JM Chiang and KF Ng) who were blind to the clinicopathological data of the tumour and to the initial results of the other observer. In areas of well-preserved tissue, the staining intensity of the cell membrane or cytoplasm was evaluated using the staining of adjacent non-involved normal mucosa as the internal control for each section. Membrane expression of E-cadherin or β-catenin was considered preserved when staining of cancer cells was as strong as that of normal glands, and the proportion of positive cancer cells in each section was more than 90%. If there was identifiable positive staining in less than 10% of the cancer cells, the tumours were recorded as having a loss of E-cadherin or β-catenin membrane expression. Nuclear staining of positive cells was defined as an intense brown colour in the nucleus (Figure 2

). The pattern of nuclear staining was defined as follows: negative group, no less than 5% scattered positive cells without any clusters; focal group, positive cells clustered in focal areas, when >5%, but <50% of the nuclei were stained; diffuse, over-expressed group, positive cells distributed diffusely, homogeneously or heterogeneously, when ⩾50% of the nuclei were stained.

### APC and β-catenin gene mutational analysis

Genomic DNA from each tumour sample and corresponding normal mucosa was extracted. SSCP analysis of β-catenin exon 3 was performed using the following primer pair: exon 3, 5′-GATTTGATGGAGTTGGACATGG-3′ and 5′-TGTTCTTGAGTGAAGGACTGAG-3′. Samples were amplified through 35 cycles on a thermocycler (Perkin-Elmer) at 95°C denaturation for 50 s, 57°C annealing for 30 s and 72°C extension for 10 min. Polymerase chain reaction (PCR) was performed in a volume of 25 μl with 20 ng of genomic DNA in a PCR buffer containing 1.5 mM MgCl2, 200 μM each deoxyribonucleoside triphosphate, 5 pmol of each primer and 0.5 units of Taq polymerase (Perkin-Elmer, Branchburg, NJ, USA).

For the investigation of the APC gene, exon 15 mutations were performed using the protein truncation test (PTT) on genomic DNA as described previously (Van der Luijt, 1994). Exons 1–14 were screened by SSCP using published oligonucleotides and PCR conditions (Van der Luijt, 1994; Li *et al*, 1999). After PCR amplification, products were loaded onto 12.5% polyacrylamide gels from a GeneGel Excel 12.5/2.4 kit (Pharmacia Biotech, AB Uppsala, Sweden) and underwent electrophoresis at 20°C. The single and double strands of the PCR products were visualised by silver staining as described previously.

### Statistics

Comparative data were analysed using the Mann–Whitney *U*-test, the Pearson's correlation coefficient and the chi-square test. A two-sided *P*<0.05 was determined as statistically significant.

## RESULTS

We collected 51 colorectal carcinoma samples, including 25 ulcerative tumours and 26 polypoid tumours. The clinicopathological parameters, including age, gender, tumour stage, tumour differentiation and tumour size were comparable between the two groups, except for right colon predominance in the polypoid CRC group and significantly deeper invasion depth in the ulcerative CRC group (Table 1

**Table 1:**
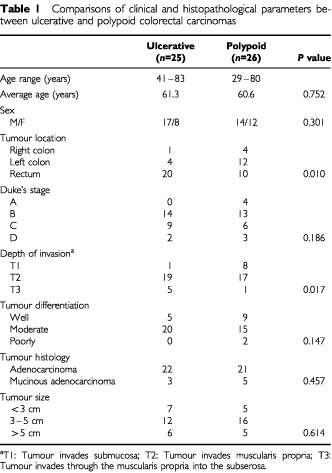
Comparisons of clinical and histopathological parameters between ulcerative and polypoid colorectal carcinomas

). The difference in depth of invasion between these two groups were mainly reflected in the smaller-sized (⩽4 cm) tumour group, while no difference in invasion depth was found for the larger-sized (>4 cm) tumour group (Table 2

**Table 2:**
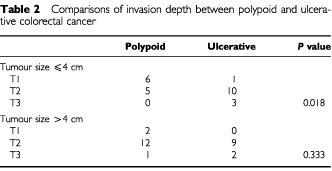
Comparisons of invasion depth between polypoid and ulcerative colorectal cancer

).

Regarding β-catenin and E-cadherin expression by immunohistochemical staining, loss of membrane staining was observed in 23 out of the 51 (45%) and 22 out of the 51 (43%) cases, respectively. Forty-nine per cent (25 out of 51) of cases showed nuclear β-catenin expression and always showed a reciprocal loss of membrane staining except for two preserved cases (Table 3

**Table 3:**
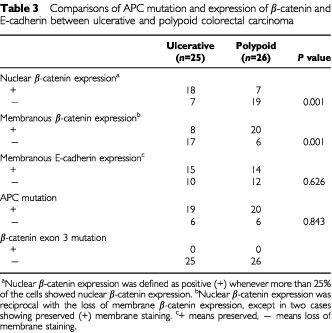
Comparisons of APC mutation and expression of β-catenin and E-cadherin between ulcerative and polypoid colorectal carcinoma

). There was no significantly different expression of E-cadherin found between polypoid and ulcerative groups. However, a significant difference in membrane β-catenin staining was found between ulcerative (17 out of 25, 68%) and polypoid (six out of 26, 23%) groups. A significant difference in nuclear expression of β-catenin (*P*=0.001) was also observed between the ulcerative (18 out of 25, 72%) and polypoid (seven out of 26) groups of CRC (Table 3) when we defined more than 25% of cells with nuclear β-catenin expression as positive, and negative was defined as expression in less than 25% of the cells. Nonetheless, nuclear β-catenin expression was typically heterogenous; we, therefore, analysed the extent of nuclear β-catenin expression with relation to these two distinct morphological types of CRC. We found a significant difference between polypoid and ulcerative types of CRC. However, we did not find a significant difference related to the depth of invasion or the location of the tumour (Table 4

**Table 4:**
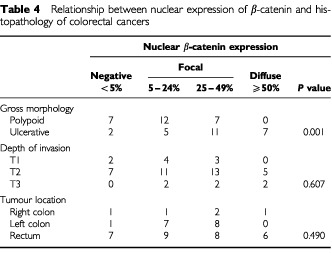
Relationship between nuclear expression of β-catenin and histopathology of colorectal cancers

).

**Figure 3:**
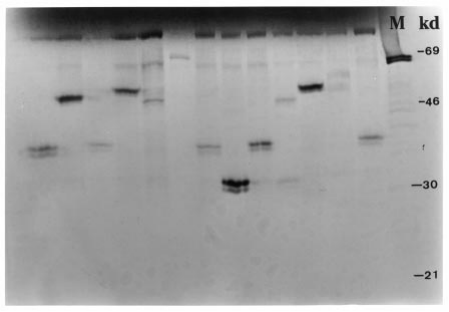
Protein truncation test for APC. Each lane represents the *in vitro* protein synthesis results of patients with sporadic colon cancer. Note that in several patients extra bands can be seen due to varied sized truncated protein. Size-fractionated on a 10% SDS-polyacrylamide gel. Lane M is luciferase as molecular weight marker. Size marker (right) is in kilodaltons (kd).

The frequency of APC mutations was 39 out of 51 (76%) cases. Among the 39 mutations found, 34 were detected in exon 15 truncated proteins (Figure 3

), two mutations were found in exon 12, one in exon 10 and one in exon 6. Nineteen out of 25 (76%) APC mutations were found in polypoid tumours and 20 out of 26 (77%) were found in ulcerative tumours (Table 3). No β-catenin exon 3 mutation was detected in this study.

Nuclear β-catenin expression was analysed in relation to its related factors. There were no correlations found among nuclear β-catenin expression and APC gene mutations (Table 5

**Table 5:**
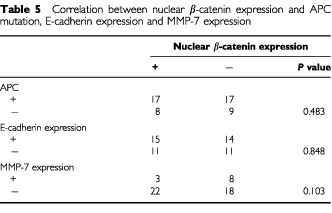
Correlation between nuclear β-catenin expression and APC mutation, E-cadherin expression and MMP-7 expression

). In addition, loss of E-cadherin expression was not significantly related to nuclear β-catenin expression. Twenty-two per cent (11 out of 51) of the cases demonstrated positive matrilysin (MMP-7) staining, and were observed in all eight mucinous colorectal carcinomas, one in poorly differentiated CRC and the other two in well differentiated CRC. Nonetheless, of these 11 cases, only three demonstrated nuclear β-catenin expression.

## DISCUSSION

We clearly demonstrated that nuclear β-catenin expression was significantly related to ulcerative growth of CRC. This result is comparable to results in a recent report (Aust *et al*, 2001) describing altered distribution of β-catenin in ulcerative colitis-related colorectal cancer.

Morphologically, the gross appearance of advanced sporadic CRC is quite variable. It is usually difficult to categorise all tumours into either the polypoid or ulcerative groups because intermediate or mixed types showing variable amounts of both components can be found. Other less frequently encountered types are flat or plateau tumours or the rare pipe-like shaped (linitis plastica) tumours. This striking diversity in the gross appearance of CRC may reflect the underlying chaotic genetic instability. In our study, we investigated purely polypoid and purely ulcerative tumours. The implications of our findings are further discussed below.

Contradictory to previous studies showing that K-*ras* codon 12 mutation is selectively related to polypoidal growth of CRC (Yamagata *et al*, 1994; Chiang *et al*, 1998), our study indicated that there were different combinations of genetic alterations occurring in morphologically different tumours during colorectal carcinogenesis. Therefore, reports trying to define the sequence or the specific sites of genetic alterations during multi-step colorectal carcinogenesis should be very carefully considered because the same combinations of genetic alterations may not always be accumulated among the morphological types of CRC. Our findings highlight that future development of responsible genes for gene therapy or genetic diagnosis for CRC may need to be individualised.

Although many *in vitro* studies using cell lines have proven that mutations in the APC tumour suppressor gene occurs in most colorectal cancer and leads to the activation of β-catenin (Munemitsu *et al*, 1995; Morin *et al*, 1997), this probably is not always the case *in vivo*. In our study, we showed that nuclear β-catenin expression was independent of APC tumour suppressor gene mutation, as was reported previously (Kobayashi *et al*, 2000). Furthermore, we did not find any β-catenin mutation itself. The question follows, therefore, what regulates or forces the nuclear translocation of β-catenin? Other genetic or epigenetic events may be present for modulating nuclear translocation. Tyrosine phosphorylation of β-catenin by a biochemical molecule such as intestinal trefoil factor has been reported (Liu *et al*, 1997). While, retinoic acid (RA) has been shown to decrease the activity of the β-catenin- lymphoid enhancer binding factor/T-cell factor signalling pathway. RA activity was also independent of APC tumour suppression and ubiquitination-dependent degradation of cytoplasmic β-catenin (Easwaran *et al*, 1999).

Although a significant relationship exists between ulcerative CRC and nuclear β-catenin expression, we did not find a significant increase in the extent of nuclear β-catenin expression related to the depth of invasion (Table 4). These findings indicate that although nuclear β-catenin expression may determine the ulcerative growth pattern of CRC, the depth of invasion may be determined by several other factors, and might be the result of a more complex process between tumour and stroma interaction. This finding also implies that higher amounts of nuclear β-catenin expression are probably necessary for the ulcerative growth from the early stage of tumour progression, while lower amounts of nuclear β-catenin expression may be sufficient to induce polypoid tumour growth (Brabletz *et al*, 2000). This finding supports a previous observation showing that β-catenin occurred in the highest concentrations in the invading line of endophytic growth of tumour cells. (Brabletz *et al*, 1998). Although we found a significant correlation between nuclear beta-catenin expression and ulcerative growth and also observed that most of the ulcerative tumours were rectal carcinomas (Table 1), we did not observe a significant relationship between tumour localisation and nuclear on tumour localisation. Furthermore, the small number of cases in this study limited further analysis of whether there are different carcinogenesis pathways in the colon and rectum.

Precisely how nuclear β-catenin expression confers ulcerative or endophytic growth to CRC remains poorly understood. Further analyses of several downstream factors of the nuclear β-catenin/TCF complex, including c-MYC (He *et al*, 1998), cyclin D1 (Tetsu and McCormick, 1999), gastrin (Koh *et al*, 2000), PPARS (He *et al*, 1999) MMP-7 (Brabletz *et al*, 1999; Crawford *et al*, 1999) are warranted. Some factors such as c-*myc*, cyclin D and gastrin reportedly relate to tumour proliferation, while others are related to tumour invasion. MMP-7 is regulated by β-catenin expression (Brabletz *et al*, 1999) and is a proteolytic enzyme related to tumour invasion. It may well be reasonable to relate proteolysis to ulcerative growth. However, in our study, we did not observe parallel expression between β-catenin and MMP-7, which is the case in cell culture studies. These negative findings may be explained similarly to other down-regulators such as tumour growth factor (TGF)-β, which is involved in a more complex process *in vivo* than *in vitro* (Gaire *et al*, 1994).

Finally, the finding that nuclear β-catenin expression is closely related to ulcerative growth of CRC further supports a previous study that showed analogies between embryonic gastrulation and β-catenin expression (Kirchner and Brabletz, 2000). Strong, diffuse β-catenin nuclear expression was observed as necessary for mesenchymal transition of tumour cells that expressed invasion behaviour, while weak nuclear β-catenin expression was only enough for epithelial transitions responsible for cell proliferation (Kirchner and Brabletz, 2000). The connection of our data and the data from embryonic development, thus, supports the concept that tumorigenesis has the properties of a complex developmental disorder (Dean, 1998).

In summary, we observed that the different combinations of genetic alterations may selectively underlie different types of CRC. Nuclear β-catenin expression is related to the ulcerative growth patterns of CRC. Although the precise mechanism remains poorly understood, its expression is independent of APC mutation. These observations further highlight the heterogenous nature of CRC, which should be kept in mind when developing a new gene therapy or a new genetic diagnosis for CRC.
